# Combined impact of TiO_2_ nanoparticles and antibiotics on the activity and bacterial community of partial nitrification system

**DOI:** 10.1371/journal.pone.0259671

**Published:** 2021-11-15

**Authors:** Han Xu, Binghua Liu, Wenyu Qi, Meng Xu, Xiaoyu Cui, Jun Liu, Qiang Li

**Affiliations:** 1 College of Agriculture and Forestry Science, Linyi University, Linyi, Shandong, China; 2 State Key Laboratory of Microbial Technology, Shandong University, Shandong, China; South China University of Technology, CHINA

## Abstract

The effects of TiO_2_ nanoparticles (nano-TiO_2_) together with antibiotics leaking into wastewater treatment plants (WWTPs), especially the partial nitrification (PN) process remain unclear. To evaluate the combined impact and mechanisms of nano-TiO_2_ and antibiotics on PN systems, batch experiments were carried out with six bench-scale sequencing batch reactors. Nano-TiO_2_ at a low level had minimal effects on the PN system. In combination with tetracycline and erythromycin, the acute impact of antibiotics was enhanced. Both steps of nitrification were retarded due to the decrease of bacterial activity and abundance, while nitrite-oxidizing bacteria were more sensitive to the inhibition than ammonia-oxidizing bacteria. *Proteobacteria* at the phylum level and *Nitrosospira* at the genus level remained predominant under single and combined impacts. The flow cytometry analysis showed that nano-TiO_2_ enhanced the toxicity of antibiotics through increasing cell permeability. Our results can help clarify the risks of nano-TiO_2_ combined with antibiotics to PN systems and explaining the behavior of nanoparticles in WWTPs.

## Introduction

In addition to traditional nitrification processes, recent studies have investigated partial nitrification (PN) as a nitrogen removal method [1]. In PN, ammonia is oxidized into nitrite by ammonia-oxidizing bacteria (AOB) without further transformation into nitrate by nitrite-oxidizing bacteria (NOB), resulting in nitrite accumulation. The accumulated nitrite can be directly converted into dinitrogen gas through denitrification, such as in the single-reactor high-activity ammonia removal over nitrite process [2]. Nitrite accumulated through PN, together with ammonia, can also be converted into dinitrogen gas under anoxic conditions by anammox bacteria, such as in the oxygen-limited autotrophic nitrification-denification process [3]. In addition, PN has attracted interest because of its several advantages over traditional nitrification such as reduced energy consumption, shorter reaction time, and lower sludge production [1].

PN is sensitive to various conditions, such as acidity, low dissolved oxygen, high salinity, heavy metals, phenols, and chloramines. Previous studies have shown that all of these factors can inhibit PN and alter the bacterial community [4–9]. In recent years, new types of inhibitors, such as antibiotics and nano-materials are being deposited into municipal and industrial wastewater treatment plants (WWTPs).

Antibiotics, commonly used antimicrobials in human being and animal husbandry, are consumed at a rate of 200,000 tons per year worldwide. Most antibiotics are discarded without transformation or degradation, resulting in numerous environmental and health problems [10]. Tetracycline is a tetracycline antibiotic and one of the most commonly used antibiotics in animal production [11]. Erythromycin, a representative macrolide antibiotic, is widely used to treat clinical infections of soft tissue, respiratory and skin [12]. Previous reports have shown that the concentration of tetracycline and erythromycin in raw wastewater can be 25–85 mg/L [13, 14]. These two antibiotics can inhibit bacterial growth and metabolism by disturbing protein synthesis. It has been reported that these two antibiotics can repress the ammonia oxidizing activity of AOB and alter the kinetics of nitrification [15].

Nano-materials are used in coating, clothing, water treatment and as catalysts and biosensors. TiO_2_ nanoparticles (nano-TiO_2_) are among the most widely used nano-materials worldwide, with a global production of up to 10,000 tons per year [16]. The predicted concentrations of nano-TiO_2_ in aeration tanks of WWTPs were in the range of 0.122±0.023 to 12.297±1.707 mg/L [17]. High concentrations of nano-TiO_2_ can inhibit both gram-positive and gram-negative bacteria (including AOB) via oxidative stress, coordination effects, and DNA damage [18–20].

Several studies have investigated the impact of single factors, such as antibiotics, on the bacterial community during PN [15, 21]. Inhibitors always coexist in the environment to affect the activated sludge system, exhibiting four main effects: independent, additive, synergistic, and antagonistic [22]. For instance, oxytetracycline and Cu(II) had antagonistic combined toxicity towards anammox activity caused by oxytetracycline-metal ion complexes [23]. In contrast, roxithromycin and Cu(II) presented synergistic toxicity because their target sites were independent [24]. In addition, the combined effect of chloramine and copper has also been proved to fit a non-competitive inhibition model [25].

Therefore, nano-TiO_2_ combined with other inhibitors should be considered as a potential risk to wastewater treatment systems, but the impacts of this multiple contamination have not been widely reported. In this study, PN batch experiments were carried out using six bench-scale sequencing batch reactors (SBRs). Nano-TiO_2_, tetracycline, and erythromycin were added at possible concentrations in WWTPs. Ammonia removal efficiency and nitrite accumulation rate were measured and calculated. The abundance and diversity of bacterial communities under the combined impacts were investigated by quantitative PCR and high-throughput sequencing. The flow cytometry analysis was carried out to identify the influence of nanoparticles and antibiotics on cell permeability.

## Materials and methods

### Activated sludge

Activated sludge was collected from Linyi College-Town Wastewater Treatment Plant in Shandong, China. The systems were operated under anaerobic–anoxic–oxic (A^2^/O) processes and the sludge was sampled from the aerobic tank. Samples were taken in sterile polyethylene bottles to the laboratory, and inoculate instantly.

### Batch experiments

The batch experiments were carried out with six bench-scale SBRs of 2 L working volume, and the schematic diagram was shown in S1 Fig. The volumetric exchange ratio was 50% and HRT was controlled at 24 h, with 11 h for the aerobic running stage, 0.5 h for the precipitate stage, and 0.5 h for the medium-change stage, respectively. The temperature and aeration rate were controlled at 30°C and 0.2 m_3_/h.

Activated sludge was inoculated into the reactor with the MLSS concentration of 3500 mg/L. After inoculation, synthetic wastewater without Nano-TiO_2_ and antibiotics was fed into the reactors for about 20 days to attain partial nitrification with the optimal operational conditions to obtain 50% ammonium and 50% nitrite. The composition of synthetic wastewater fed to the reactor was as follow (g/L): (NH_4_)_2_SO_4_ 4.0 g (approximately 400 mg/L), Na_2_CO_3_ 1.0, NaCl 1.0, K_2_HPO_4_·3H_2_O 1.0, MgSO_4_·7H_2_O 0.05, CaCl_2_ 0.1, pH 8.0 [26].

### Chemical analysis and calculations

DO and pH were measured by electrode method. Ammonia nitrogen (NH_4_^+^-N), nitrate nitrogen (NO_3_^-^N) and nitrite nitrogen (NO_2_^-^N) were analyzed according to standard methods for the examination of water and wastewater [27]. Ammonia removal efficiency (ARE) and nitrite accumulation rate (NAR) were calculated as Eqs (1) ~ (2).


$$\text{ARE}=\left(\;{\left[{\text{NH}}_{4}{}^{+}-\text{N}\right]}_{\text{influent}}-{\left[{\text{NH}}_{4}{}^{+}-\text{N}\right]}_{\text{effluent}}\right)/\text{time}$$ (1)

$$\text{NAR}={\left[{\text{NO}}_{2}{}^{-}-\text{N}\right]}_{\text{effluent}}/({\left[{\text{NO}}_{2}{}^{-}-\text{N}\right]}_{\text{effluent}}+{\left[{\text{NO}}_{3}{}^{-}-\text{N}\right]}_{\text{effluent}})$$ (2)

### Preparation and addition of Nano-TiO_2_ and antibiotics stock solutions

Anatase nano-TiO_2_ with a particle size of 25±10 nm and a specific area of 20–80 m^2^/g were acquired from Sigma-Aldrich Co. Nanoparticles were added into ultrapure water as the concentration of 1 g/L and treated with ultrasonic wave at 33 W for 30 min to scatter it equably. The stock solutions of tetracycline and erythromycin were also made with ultrapure water as the concentration of 1 g/L and then stored at 4 °C.

After attaining partial nitrification, three kinds of Nano-TiO_2_, tetracycline and erythromycin stock solutions were added into influent respectively or together, to keep the initial doses as Table 1. These concentrations were selected in agreement with previous descriptions in wastewater influents [17].

**Table 1:** The concentration of Nano-TiO_2_ and antibiotics in the influents of each reactor.

	Control	Nano	Tet	Nano&Tet	Ery	Nano&Ery
nano-TiO_2_ (mg/L)	/	10	/	10	/	10
tetracycline (mg/L)	/	/	50	50	/	/
erythromycin (mg/L)	/	/	/	/	50	50

### DNA extraction from the sludge samples

The sludge samples were taken from the reactor in every phase, including the initial sludge sample written as 0. Genomic DNA was extracted in triplicate from all sludge samples using a SoilGen DNA Kit (CWBIO) according to the manufacturer’s instructions. DNA integrity was checked by 0.8% agarose gel electrophoresis. The purity and the quantity of extracted DNA were determined by UV spectrophotometry at 260 and 280 nm. DNA extracts were stored at –20°C.

### Quantitative PCR for AOB, NOB and total bacteria

Quantification of AOB, NOB and total bacteria was performed using the primers CTO189R/CTO654R, NSR-1113F/NSR-1264R and 338F/518R. All the information of the primers was listed in S1 Table. The qPCR was carried out with 25 μl volume in eight-tubes-per-strip (Axygen) and 2× Power SYBR^®^ Green PCR Master Mix (Takara) was used. All PCR amplification used the same thermocycling steps as follows: 5 min at 95°C, followed by 40 cycles of 60 s at 95°C, 45 s at 57°C and 45 s at 72°C, and finally 10 min at 72°C [28]. The standard DNA was diluted to 1.47×10^5^−1.47×10^10^ (total bacteria 16S rDNA), 5.02×10^4^−5.02×10^9^ (AOB 16S rDNA), and 8.04×10^4^−8.04×10^9^ (NOB 16S rDNA) copies/μl, respectively. The reaction was carried out and monitored with an iCycler iQ^™^ Real-Time PCR Detection System (Bio-Rad). The threshold cycle (Ct) values were measured after amplification and the copy numbers were calculated using the standard curve [29].

### High-throughput sequencing and bioinformatics analysis

The 515F with barcode and 907R primers (as S1 Table) were used to amplify the V4 region of 16S rRNA genes and the thermocycling steps were as follow: 5 min at 95°C, followed by 30 cycles of 30 s at 95°C, 30 s at 55°C and 45 s at 72°C, and finally 10 min at 72°C. Amplicons were extracted from 2% agarose gels and purified using the AxyPrep DNA Gel Extraction Kit (Axygen, USA) according to the manufacturer’s instructions. Purified amplicons were used for sequencing on Illumina MiSeq PE300 platform (Illumina, USA) using standard protocols. To minimize the effects of random sequencing errors, the completeness of the barcodes and the adapters was checked and the sequences shorter than 50 bps were removed. The sequence number of each sample was normalized and the trimmed sequences for each operational taxonomic unit (OTU) with a 97% sequence identity were obtained using Usearch V7.1. The numbers of sequences and operational taxonomic units (OTUs) were counted as S2 Table, which were all above 31000 and 1100 except the initial sludge. All sequences have been submitted to the National Center Biotechnology Information at accession number PRJNA749958.

Taxonomy was assigned via the RDP classifier V2.2 with the Silva databases (http://www.arb-silva.de). Rarefaction curves and Alpha-diversity analysis were generated by Mothur V1.3. Hierarchical cluster analysis, principal component analysis (PCA), and community heatmap analysis were performed to depict the similarity and difference between the communities in each sample by R packages.

### Flow cytometry analysis for cell permeability

Differential centrifugation was used to obtain bacterial suspension from activated sludge samples, firstly at 4000 rpm for supernatant and then at 10000 rpm for precipitation. After washed twice with 0.85% (w/v) NaCl solution, the bacterial suspension was diluted to a cell density with an optical density at 600 nm (OD_600_) of 0.10±0.02 [30]. One mL of bacterial suspension was stained with 10 μl of propidium iodide (PI) (1 mg/ml, OMEGA, USA) and incubated in the dark for 8 min before measurement [31, 32]. The cell permeability was tested by flow cytometry analysis (FCM, BD FACSCalibur, USA) and PI fluorescent signal was excited by 488 nm. All data were processed with the CellQuest Pro.

## Results and discussion

### Achievement of partial nitrification

The batch experiments were carried out with six bench-scale SBRs. Ammonium, nitrite, and nitrate were measured and ammonia removal efficiency and nitrite accumulation rate were calculated.

**Fig 1:**
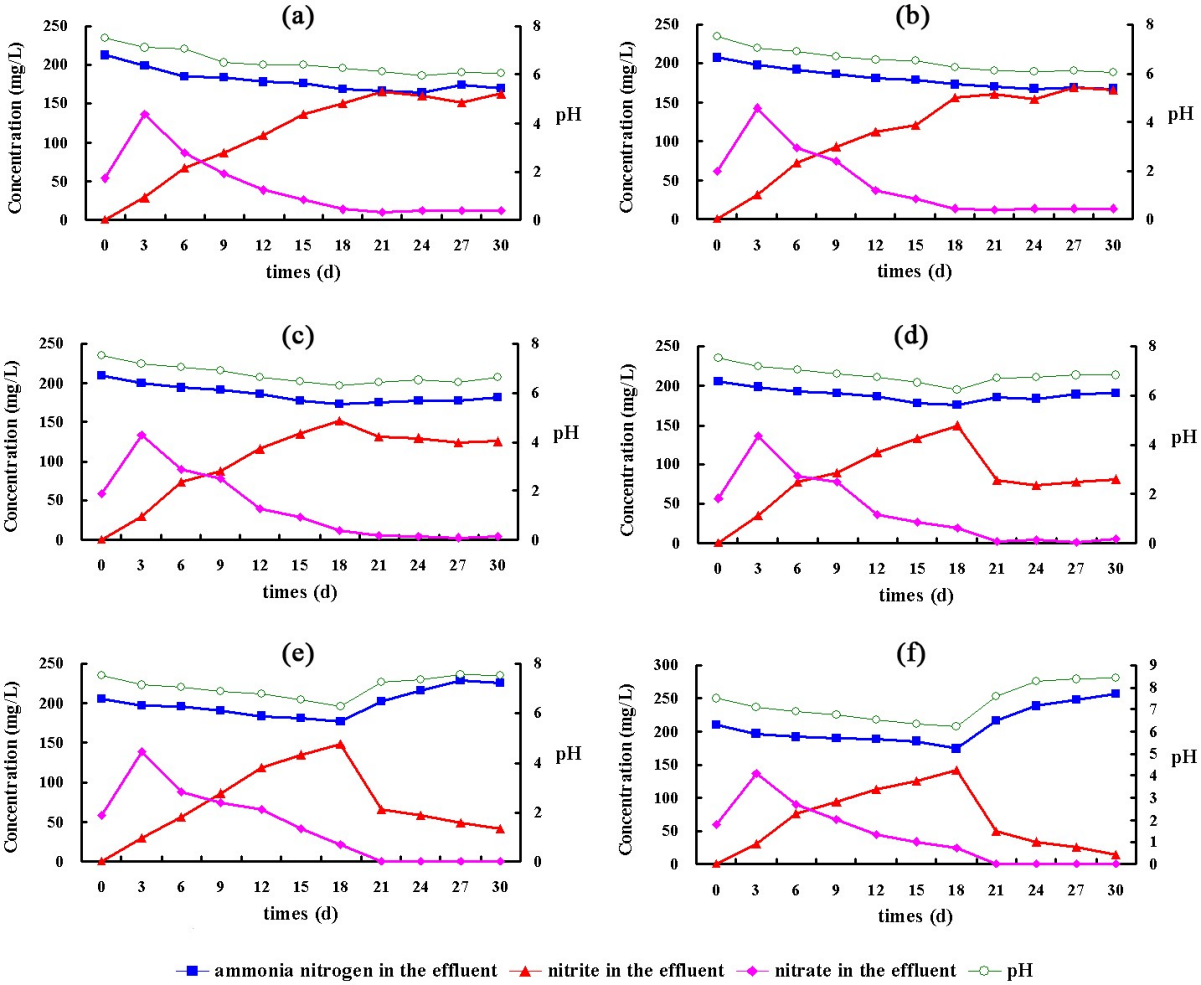
Ammonia nitrogen, nitrite and nitrate concentrations in all systems. (a) Control group, (b) Nano group, (c) Tet group, (d) Ery group, (e) Nano&Tet group, (f) Nano&Ery group.

After the reactors were started, the pH and concentration of ammonia nitrogen in the effluent continually decreased, demonstrating the enhancement of the ammonia oxidation reactions. The concentration of nitrate decreased after the 3^rd^ day and nitrite accumulated. Then PN was accomplished until the 20^th^ day in each reactor. The ammonia removal efficiency and nitrite accumulation rate reached approximately 9.58 mg/L/h and 93.2%, respectively, and the ratio between NH_4_^+^-N and NO_2_^-^N was approximately 1:1 (Fig 1a).

Previous studies have shown that NOB is sensitive to free ammonia (FA) and 0.1–1.0 mg/L FA can inhibit NOB growth [33, 34]. AOB has exhibited a stronger tolerance to FA than NOB and can tolerate 300 mg/L FA [35]. Therefore, the system continued PN condition under high FA stress and 27.3 mg/L of FA was accumulated, which was calculated as equation (S1 in S1 Appendix) based on the concentration of ammonia nitrogen and pH value (approximately 6.0).

### Combined impact of TiO_2_ nanoparticles and antibiotics

After achieving PN, nano-TiO_2_, tetracycline, and erythromycin were added to the synthetic wastewater at the concentrations shown in Table 1.

In the Nano group (Fig 1b), only nano-TiO_2_ was added. Ammonia removal efficiency and nitrite accumulation rate were similar to those of the control group (Fig 1a), demonstrating that a low level of nano-TiO_2_ did not inhibit the PN system. Previous studies have shown that nano-TiO_2_ levels above 20 mg/g in the soil and above 50 mg/L in activated sludge systems can negatively impact environmental bacterial communities [19, 20]. Other studies found that metal oxide nanoparticles were harmless at concentrations predicted to be environmentally relevant and did not inhibit the ammonia oxidation rate or the AOB community [36].

In the Tet group (Fig 1c), only tetracycline was added. Ammonia removal efficiency decreased to 9.09 mg/L/h and nitrite accumulation rate reached 96.4%. Both nitrification steps were completely inhibited and slowed by tetracycline. However, NOB were more sensitive to tetracycline than were AOB, and the inhibition of tetracycline to NOB was found to be stronger than that to AOB [37].

In the Ery group (Fig 1d), only erythromycin was added. Ammonia removal efficiency decreased to 7.27 mg/L/h, indicating that the metabolism of AOB was inhibited. Nitrite oxidation was completely blocked by erythromycin and a significantly low concentration of nitrate was detected in the effluent. The tolerance of AOB and NOB to erythromycin was weaker than that to tetracycline. After adding erythromycin for approximately 10 days, the activated sludge disintegrated and the reactor had to be stopped.

In the Nano&Tet and Nano&Ery groups (Fig 1e and 1f), nano-TiO_2_ was added to the reactors together with tetracycline and erythromycin, respectively. Ammonia removal efficiency decreased to 8.72 and 5.98 mg/L/h, respectively, which are lower than the values for the Tet and Ery groups. The nitrite accumulation rate reached 94.5% and 100%, respectively. This indicated that nano-TiO_2_ enhanced the acute impact of antibiotics. Both short-term exposure of nano-TiO_2_ at high concentration (50–100 mg/L) and long-term exposure of nano-TiO_2_ at low concentration (5–10 mg/L) negatively impacted the operation of activated sludge system, including inhibition of the organic matter, nitrogen and phosphorus removal, reduction of oxygen utilization, promotion of reactive oxygen species production and lactate dehydrogenase release [38, 39]. In contrast, short-term exposure of nano-TiO_2_ at low concentration could not significantly affect the system behavior, but it might enhance the toxicity of coexisting pollutants, such as heavy metals and natural organic matters [40, 41].

### The abundance of nitrifiers and bacteria

**Fig 2:**
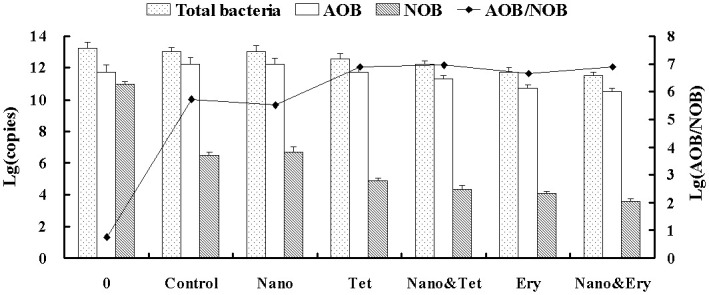
The count of AOB, NOB and total bacteria measured by qPCR.

To investigate the abundance of the bacterial community under different conditions, quantitative PCR was used to measure the abundance variations in AOB, NOB and total bacteria, and the results were shown in Fig 2.

The abundance of AOB was 5.74 × 10^11^ copies/dry sludge (DS) in the initial sludge, accounting for 3.1% of total bacteria. The autotrophic and ammonia-rich environment was beneficial for the enrichment of AOB, which increased to 1.75 × 10^12^ copies/DS and 15.9% of total bacteria when PN was obtained. Simultaneously, the abundance of total bacteria decreased fractionally to 1.10 × 10^13^ copies/DS from 1.85 × 10^13^ copies/DS, as the abundance of autotrophic bacteria increased slowly for its low growth rate while the abundance of heterotrophic bacteria deceased fast in autotrophic system without carbon source. The ratio of AOB and NOB was enhanced to 5.37 × 10^5^ because NOB was restrained by FA. The specific growth rate of AOB was about 6.3 fold of NOB under high FA stress (S2 Table), leading to the large gap in bacterial count between AOB and NOB.

In the Nano group, the abundances of AOB, NOB, and total bacteria were all similar to those in the control group, further demonstrating that nano-TiO_2_ had only a minimal impact on the PN system at the predicted concentration in WWTPs.

In the Tet group and Ery group, the presence of tetracycline or erythromycin in the synthetic wastewater had an inhibitory effect on both AOB and NOB. While NOB was more susceptible to antibiotics, so that its decrease was larger than that of AOB and the increase in growth rate between AOB and NOB became larger under antibiotics treatment. The abundances of AOB and NOB were 5.88 × 10^11^ and 7.62 × 10^4^ copies/DS in the Tet group and 5.26 × 10^10^ and 1.16 × 10^4^ copies/DS in the Ery group, respectively. These results demonstrated that compared to tetracycline, erythromycin inhibited nitrifiers more strongly, which is consistent with the results of Katipoglu-Yazan et al. [15]. The decrease in total bacteria was small because several types of heterotrophic bacteria exhibited antibiotic resistance.

Antibiotics combined with nano-TiO_2_ showed greater toxicity towards nitrifiers, reducing the abundance of AOB and NOB to 2.02×10^11^ and 2.28×10^4^ copies/DS in the Nano&Tet group and 3.11×10^10^ and 3.88×10^3^ copies/DS in the Nano&Ery group. The cytotoxicity and environmental risk of nano-TiO_2_ has been widely reported [19]. Nanoparticles could accumulate on the surface of cells when approaching bacterial communities [42]. This can lead to oxidative stress or nucleic acid damage, even if only a small quantity of nanoparticles enters the cells [43]. This damage can also be combined with the negative effects of antibiotics. Furthermore, nanoparticles adhered to the surface of bacterial cells can change the structure and properties of the cell membrane to enhance cellular permeability [44], which allows a greater amount of inhibitors to pass through the cell membrane.

### The diversity of bacterial community

The Chao estimator and ACE estimator were calculated to characterize community richness, whereas the Shannon index and Simpson index were calculated to estimate community diversity (Table 2). The lowest richness and diversity of the bacterial community was observed in the initial sludge, which showed that PN could enhance both richness and diversity of bacterial community in comparison with traditional activated sludge process. After PN was obtained, the community richness and diversity showed a remarkable increase, but nano-TiO_2_ did not influence this richness and diversity. Compared to the control group, erythromycin shock made the community richness, diversity and evenness lower, which implied that erythromycin washed out a large number of microorganisms. In contrast, tetracycline increased the evenness, which implied that tetracycline uniformized bacterial community. This might be due that tetracycline reduced the relative proportions of the dominant species in the microbial community, which could leave niche for rare species. Similar results were also reported by previous studies [45]. Therefore, the uniformized microbial community caused by tetracycline also showed higher richness and diversity compared to the control treatment. Combined with nano-TiO_2_ could made the community diversity a tiny drop in both tetracycline and erythromycin groups.

**Table 2:** The alpha diversity index of each experimental group.

	Chao	ACE	Shannon	Simpson	Evenness
0	1066.05	1045.38	4.37	0.060	0.651
Control	1418.78	1411.15	4.80	0.039	0.678
Nano	1422.91	1407.19	4.84	0.037	0.684
Tet	1473.50	1461.43	5.13	0.021	0.727
Nano&Tet	1461.62	1425.38	5.03	0.026	0.710
Ery	1398.60	1399.26	4.73	0.054	0.671
Nano&Ery	1379.04	1367.56	4.68	0.057	0.660

**Fig 3:**
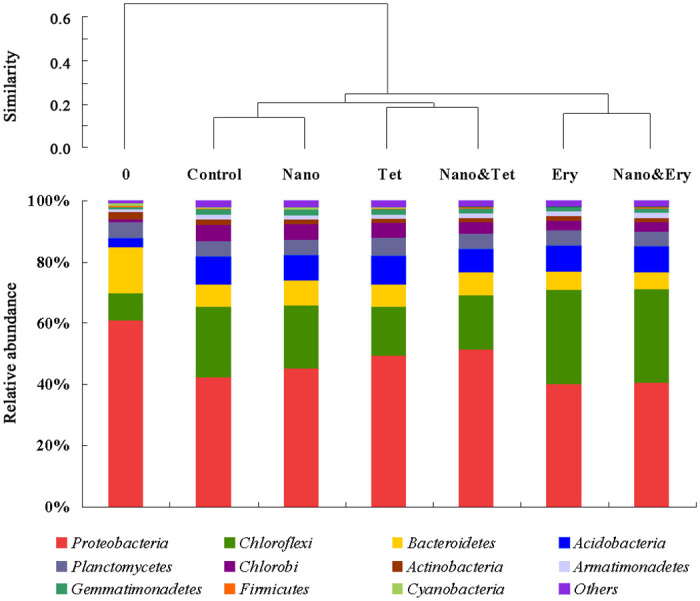
Relative abundance and hierarchical cluster analysis of all samples at phylum level.

Several phyla remained predominant in all experimental groups, including *Proteobacteria*, *Chloroflexi*, *Bacteroidetes*, *Acidobacteria*, *Planctomycetes*, and *Chlorobi* (Fig 3). AOB and NOB were affiliated with *Proteobacteria*, the most dominant phylum accounting for 35.8–59.1% of organisms in each system. The abundance of *Proteobacteria* in the Tet group was higher than that in the control group. The amounts of *Chloroflexi*, *Acidobacteria*, and *Chlorobi* enhanced after autotrophic domestication. It has been reported that these three phyla are frequently detected in PN systems [5, 46, 47] and several members of them are identified as autotrophic bacteria [48]. In addition, the abundance of *Chloroflexi*, the second most dominant phylum, increased under erythromycin shock.

**Fig 4:**
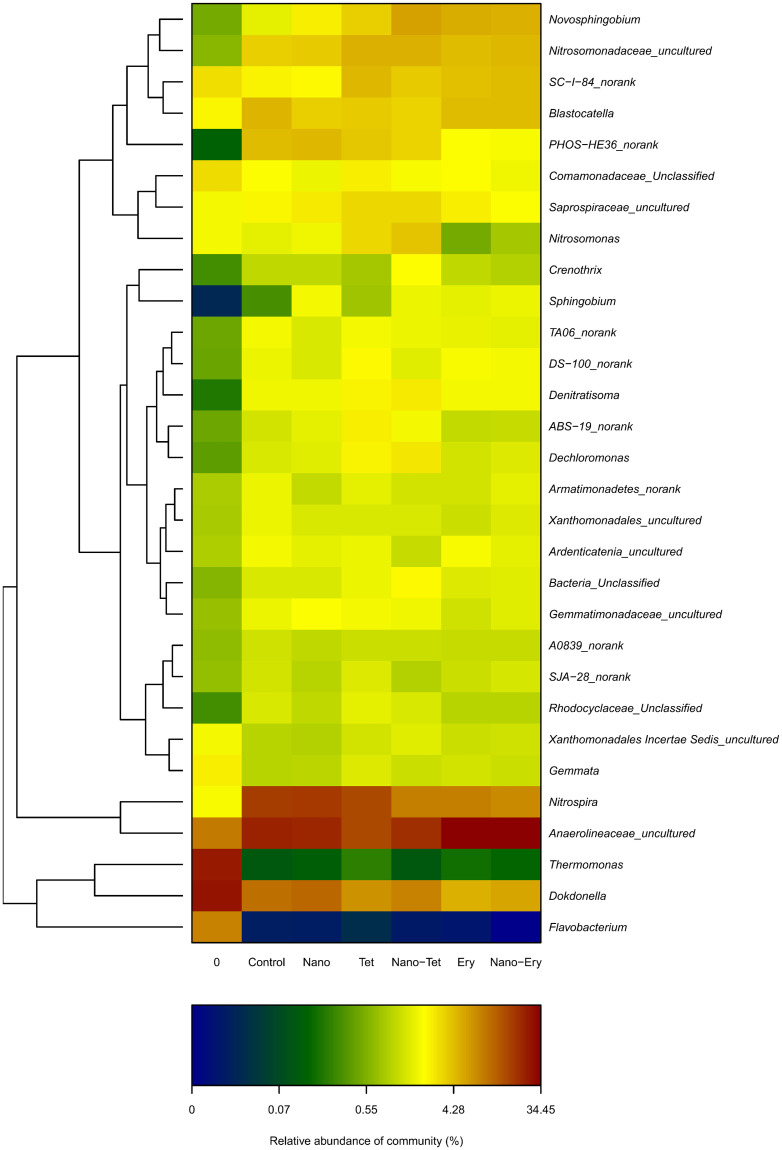
Bacterial community heatmap analysis of all samples at genus level. Top 30 genera were selected and the color intensity of scale indicates relative abundance of each OTU read.

Although the predominant phyla remained stable, the predominant genera shifted between different experimental groups (Fig 4). *Nitrosomonas* and *Nitrosospira*, the most important AOB in activated sludge, showed similar richness accounting for approximately 1.3% of bacteria in the initial sludge. After PN was achieved, *Nitrosospira* became one of the predominant genera at an abundance of more than 12%, but decreased under antibiotic shock, reaching the lowest value in the Nano&Ery group. *Nitrosomonas* showed a relative abundance of 1/5 to 1/10 lower than *Nitrosospira* in all PN systems, but increased under tetracycline shock. AOB has been reported to include several different species, such as the strains of *Nitrosococcus* sp. within *Gamma-Proteobacteria*, and the genera *Nitrosomonas* and *Nitrosospira* within *Beta-Proteobacteria* [49]. In this study, both *Nitrosomonas* and *Nitrosospira* were enriched, but *Nitrosococcus* was not detected according to phylogenetic analysis. Previous studies have shown that high nitrogen environments may be beneficial for the enrichment of *Nitrosospira* [50, 51].

Some genera, such as *Sphingobium*, were notably enhanced after PN, while others, such as *Flavobacterium*, were washed out under high ammonia nitrogen. However, none of the top 30 predominant genera in the control group were removed under antibiotics and nanoparticle shock, suggesting that combined shock has a lower impact than autotrophic domestication under high nitrogen. The alpha-diversity index (Table 2), hierarchical cluster analysis (Fig 3), and PCA (S3 Fig) revealed that initial sludge was separated from the other groups. Additionally, the antibiotics had a stronger impact on the shift in the bacterial community than nano-TiO_2_.

### The change of cell permeability

**Fig 5:**
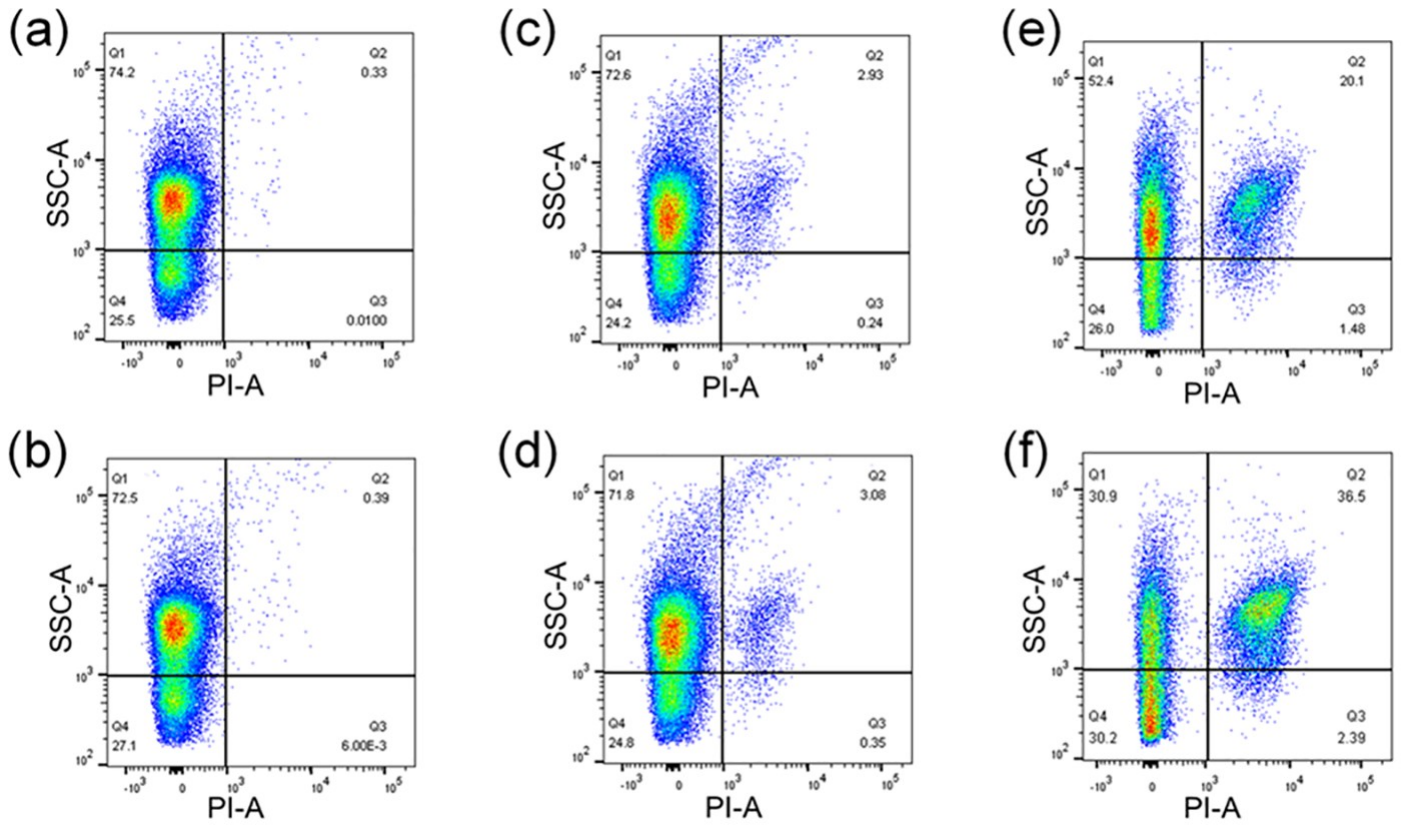
Flow cytometry analysis for bacterial cell permeability of each sludge samples. (a) Control group, (b) Nano group, (c) Tet group, (d) Ery group, (e) Nano&Tet group, (f) Nano&Ery group.

The flow cytometry analysis was used to investigate the influence of nano-TiO_2_ and antibiotics on cell permeability (Fig 5). The PI uptake in the Nano group had a tiny increase compared to the control group. Although low-dose nano-TiO_2_ presented no harm to the community in activated sludge, it enhanced bacterial cell permeability. Some other nanoparticles also showed the same function [52, 53]. Tetracycline and erythromycin inhibited the metabolism of bacterial cells and generated a mass of dead cells by binding ribosomal subunit and disturbing protein synthesis, which significantly increased the PI uptake.

In the Nano&Tet group and the Nano&Ery group, PI uptake was much higher than that in the Tet group and the Ery group, suggesting that the combined impacts significantly decreased the antibiotic resistance of the systems [31, 32]. Bacteria can reduce intracellular availability of antibiotics by inhibiting or replacing outer membrane porins and overexpressing efflux pumps. Previous studies have proved that some kinds of nanoparticles, such as silver nanoparticles (AgNPs), have synergistic antimicrobial effect with antibiotics [53]. As photocatalyst, low-dose nano-TiO_2_ lost its antimicrobial effect in dark environment, but could raise intracellular availability of antibiotics by enhancing cell permeability. Therefore, the environmental risk of nanoparticles combined with other pollutants need more attention.

## Supporting information

S1 Appendix The equations of free ammonia and specific growth rates of AOB and NOB. (DOC)

S1 Fig Schematic diagram of the SBR. 1: reaction tank; 2: storage tank; 3: pump; 4: blower; 5: air diffuser; 6: flow meter; 7: time-delay device; 8: influent; 9: effluent.
(TIF)

S2 Fig The rarefaction curve of all samples. (TIF)

S3 Fig Principal component analysis (PCA) based on Illumina sequencing data. (TIF)

S1 Table The PCR primer sequences and targets in this study. (DOC)

S2 Table Numbers of sequences and OTUs. (DOC)

S3 Table The specific growth rates of AOB and NOB. (DOC)
