# Supplementary material for: Combined impact of TiO2 nanoparticles and antibiotics on the activity and bacterial community of partial nitrification system
Source: PLoS One. 2021 Nov 15;16(11):e0259671. doi: 10.1371/journal.pone.0259671 (PMC8592496; doi:10.1371/journal.pone.0259671)
Supplement: S1 Appendix — (DOC) [file pone.0259671.s001.doc]

The concentration of free ammonia (S_FA_) was calculated as equation (S1). The specific growth rates of AOB and NOB were calculated as equation (S2) and (S3).

*S_FA_ (mg/L) =$\frac{17}{14}$×[NH_4_^+^-N]×$10^{pH}$/ (EXP$\frac{6334}{273+T}$+$10^{pH}$)* (S1)

$\mu^{AOB}$*=$\mu_{\text{max}}^{AOB}$ $\mu^{AOB}$(T) $\mu^{AOB}$(S_DO_) $\mu^{AOB}$(S_FA_) $\mu^{AOB}$(S_NH4_)*

*=*$\mu_{\text{max}}^{AOB}$ $e^{\theta_{T}^{AOB} \text{（}T\text{-}35\text{）}}$ $\frac{S_{DO}}{K_{DO}^{AOB}+S_{DO}}$ $\frac{K_{IFA}^{AOB}}{K_{IFA}^{AOB}+S_{FA}}$ $\frac{S_{NH_{4}}}{K_{NH_{4}}^{AOB}+S_{NH_{4}}}$ (S2)

$\mu^{NOB}$*=$\mu_{\text{max}}^{NOB}$ $\mu^{NOB}$(T) $\mu^{NOB}$(S_DO_) $\mu^{NOB}$(S_FA_) $\mu^{NOB}$(S_NH4_)*

*=*$\mu_{\text{max}}^{NOB}$ $e^{\theta_{T}^{NOB} \text{（}T\text{-}35\text{）}}$ $\frac{S_{DO}}{K_{DO}^{NOB}+S_{DO}}$ $\frac{K_{IFA}^{NOB}}{K_{IFA}^{NOB}+S_{FA}}$ $\frac{S_{NO_{2}}}{K_{NO_{2}}^{NOB}+S_{NO_{2}}}$  (S3)

$\mu_{\text{max}}^{AOB}$=6.60; $\theta_{\text{T}}^{AOB}$=0.120; $K_{DO}^{AOB}$=0.162; $K_{NH_{4}}^{AOB}$=1.6; $K_{IFA}^{AOB}$=3000;

$\mu_{\text{max}}^{NOB}$=1.05; $\theta_{\text{T}}^{NOB}$=0.078; $K_{DO}^{NOB}$=0.544; $K_{NO_{2}}^{NOB}$=2.78; $K_{IFA}^{NOB}$=20.
