# Supplementary figures and images for: Combined impact of TiO2 nanoparticles and antibiotics on the activity and bacterial community of partial nitrification system
Source: PLoS One. 2021 Nov 15;16(11):e0259671. doi: 10.1371/journal.pone.0259671 (PMC8592496; doi:10.1371/journal.pone.0259671)

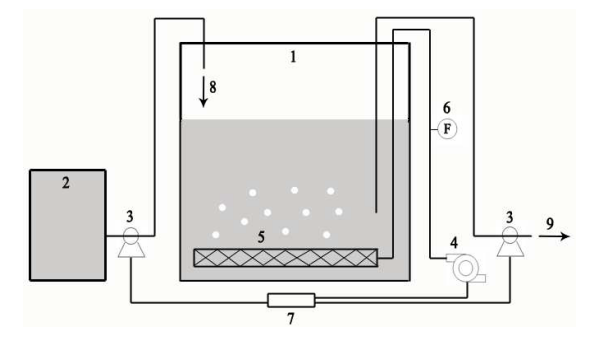

Supplement: S1 Fig — 1: reaction tank; 2: storage tank; 3: pump; 4: blower; 5: air diffuser; 6: flow meter; 7: time-delay device; 8: influent; 9: effluent. (TIF) [file pone.0259671.s002.tif]

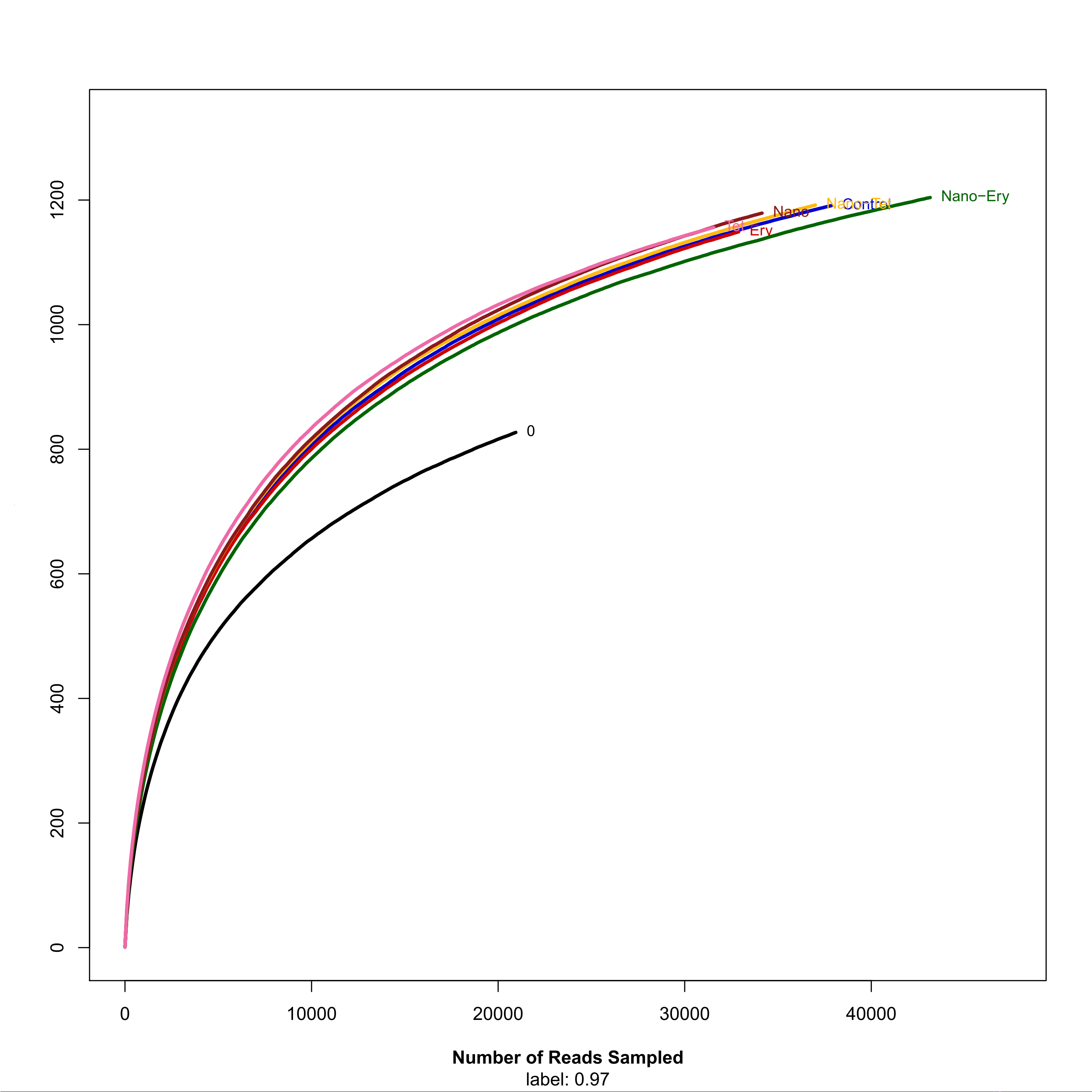

Supplement: S2 Fig — (TIF) [file pone.0259671.s003.tif]

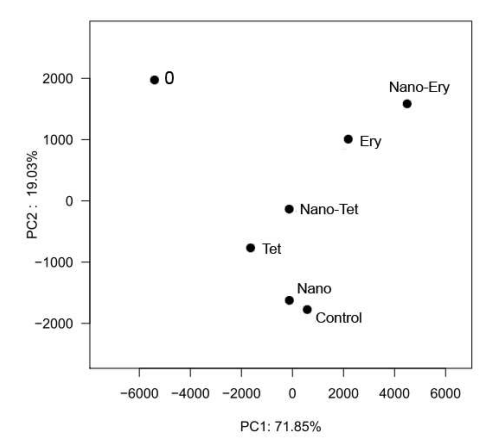

Supplement: S3 Fig — (TIF) [file pone.0259671.s004.tif]
