# Supplementary material for: Combined impact of TiO2 nanoparticles and antibiotics on the activity and bacterial community of partial nitrification system
Source: PLoS One. 2021 Nov 15;16(11):e0259671. doi: 10.1371/journal.pone.0259671 (PMC8592496; doi:10.1371/journal.pone.0259671)
Supplement: S1 Table — (DOC) [file pone.0259671.s005.doc]

Table S1 The PCR primer sequences and targets in this study.

| Primers | Primer sequence | Target (speciﬁcity) | Reference |
| --- | --- | --- | --- |
| CTO189f | GGAGRAAAGYAGGGGATCG | AOB 16S rDNA | Kowalchuk et al., 1997 |
| CTO654r | CTAGCYTTGTAGTTTCAAACGC |
| NSR-1113F | CCTGCTTTCAGTTGCTACCG | NOB 16S rDNA | Dionisi et al., 2002 |
| NSR-1264R | GTTTGCAGCGCTTTGTACCG |
| 338F | CGCCCGCCGCGCGCGGC | Total 16S rDNA | Muyzer et al., 1993 |
| 518R | ATTACCGCGGCTGCTGG |
| 515F | GTGCCAGCMGCCGCGG | Total 16S rDNA | Sui et al., 2016 |
| 907R | CCGTCAATTCMTTTRAGTTT |
