# Supplementary material for: Combined impact of TiO2 nanoparticles and antibiotics on the activity and bacterial community of partial nitrification system
Source: PLoS One. 2021 Nov 15;16(11):e0259671. doi: 10.1371/journal.pone.0259671 (PMC8592496; doi:10.1371/journal.pone.0259671)
Supplement: S2 Table — (DOC) [file pone.0259671.s006.doc]

Table S2 Numbers of sequences and OTUs.

|  | 0 | Control | Nano | Tet | Nano&Tet | Ery | Nano&Ery |
| --- | --- | --- | --- | --- | --- | --- | --- |
| No. of sequences | 20947 | 37861 | 34145 | 31555 | 37000 | 32890 | 43157 |
| No. of OTUs _0.03_ | 827 | 1191 | 1179 | 1156 | 1192 | 1149 | 1204 |

* Clustered at the 3% distance level.
