# Supplementary material for: Combined impact of TiO2 nanoparticles and antibiotics on the activity and bacterial community of partial nitrification system
Source: PLoS One. 2021 Nov 15;16(11):e0259671. doi: 10.1371/journal.pone.0259671 (PMC8592496; doi:10.1371/journal.pone.0259671)
Supplement: S3 Table — (DOC) [file pone.0259671.s007.doc]

Table S3 The specific growth rates of AOB and NOB

|  | $\mu^{AOB}$ | $\mu^{NOB}$ | $\mu^{AOB}$/$\mu^{NOB}$ |
| --- | --- | --- | --- |
| Specific growth rate (d^-1^) | 1.0793 | 0.1714 | 6.30 |

Calculated with equation (S2) and (S3).
